# Separation of early afterdepolarizations from arrhythmogenic substrate in the isolated perfused hypokalaemic murine heart through modifiers of calcium homeostasis

**DOI:** 10.1111/j.1748-1716.2007.01715.x

**Published:** 2007-09

**Authors:** M J Killeen, I S Gurung, G Thomas, K S Stokoe, A A Grace, C L-H Huang

**Affiliations:** 1 Physiological Laboratory, University of Cambridge Cambridge, UK; 2 Section of Cardiovascular Biology, Department of Biochemistry, University of Cambridge Cambridge, UK

**Keywords:** arrhythmogenesis, early afterdepolarizations, hypokalaemia, mouse heart, transmural gradients of repolarization

## Abstract

**Aims:**

We resolved roles for early afterdepolarizations (EADs) and transmural gradients of repolarization in arrhythmogenesis in Langendorff-perfused hypokalaemic murine hearts paced from the right ventricular epicardium.

**Methods:**

Left ventricular epicardial and endocardial monophasic action potentials (MAPs) and arrhythmogenic tendency were compared in the presence and absence of the L-type Ca^2+^ channel blocker nifedipine (10 nm–1 μm) and the calmodulin kinase type II inhibitor KN-93 (2 μm).

**Results:**

All the hypokalaemic hearts studied showed prolonged epicardial and endocardial MAPs, decreased epicardial-endocardial APD_90_ difference, EADs, triggered beats and ventricular tachycardia (VT) (*n* = 6). In all *spontaneously beating* hearts, 100 (but not 10) nm nifedipine reduced both the incidence of EADs and triggered beats from 66.9 ± 15.7% to 28.3 ± 8.7% and episodes of VT from 10.8 ± 6.3% to 1.2 ± 0.7% of MAPs (*n* = 6 hearts, *P* < 0.05); 1 μm nifedipine abolished *all* these phenomena (*n* = 6). In contrast programmed electrical stimulation (PES) still triggered VT in six of six hearts with 0, 10 and 100 nm but not 1 μm nifedipine. 1 μm nifedipine selectively reduced epicardial (from 66.1 ± 3.4 to 46.2 ± 2.5 ms) but not endocardial APD_90_, thereby restoring ΔAPD_90_ from −5.9 ± 2.5 to 15.5 ± 3.2 ms, close to normokalaemic values. KN-93 similarly reduced EADs, triggered beats and VT in spontaneously beating hearts to 29.6 ± 8.9% and 1.7 ± 1.1% respectively (*n* = 6) yet permitted PES-induced VT (*n* = 6), in the presence of a persistently negative ΔAPD_90_.

**Conclusions:**

These findings empirically implicate *both* EADs and triggered beats *alongside* arrhythmogenic substrate of ΔAPD_90_ in VT pathogenesis at the whole heart level.

Hypokalaemia is a recognized risk factor for the development of torsade de pointes (TdP), a life threatening form of ventricular tachycardia (VT), in which the electrocardiographic QRS complexes appear to twist about the isoelectric line (Antzelevitch *et al.*, 1996; Berthet *et al.* 1999). Although several different mechanisms of TdP induction have been reported, the two most common theories, not necessarily exclusive, are (1) Delayed repolarization, reflecting cardiac action potential duration (APD) prolongation (Gintant *et al.* 1991, Thomas *et al.* 2007a) leaves the myocardium vulnerable to cellular depolarizations occurring in phases 2 or 3 of repolarization, early afterdepolarizations (EADs), which may give rise to salvos of premature action potentials, triggered activity and subsequent episodes of VT (Eckardt *et al.* 1998, Viswanathan *et al.* 1999). EADs have been implicated as critical initiation factors for arrhythmias in the congenital long QT (LQT) syndrome (Shimizu *et al.* 1991), drug-induced LQT syndrome (Aiba *et al.* 2005) and electrolyte abnormalities such as hypokalaemia (Killeen *et al.* 2007). (2) Differential expression of cardiac ion channel currents throughout the thickness of the ventricular wall, establishes a transmural gradient of APD, which may become significantly altered following cardiac action potential prolongation. An alteration in the transmural gradient of APD, and hence refractoriness is a potentially arrhythmogenic mechanism of action (Janse and Wit, 1989).

Previous studies in isolated myocytes have thus indicated that EADs can occur in most cardiac cell types; this however, does not rule out the fact that specific groups of cells may be intricately involved in the generation of EADs. Studies of isolated myocytes or myocardial tissue preparations may therefore lack fundamental cell types that are necessary for the genesis of EADs. Single cell studies have failed to clarify the relationship between EADs at the single myocyte level and arrhythmogenesis at the whole heart level, and the relationship between EADs and transmural gradients and repolarization. Additionally, the vast majority of single cell studies did not take into consideration important transmural differences in the patterns of ion channel expression in the mammalian heart, choosing to study a mixed population of both epicardial and endocardial myocytes. The use of whole hearts permits the examination of properties of intercellularly coupled cells *in situ*, not possible in isolated cell preparations; intercellular coupling has been demonstrated to either suppress or facilitate EADs (Huelsing *et al.* 2000). Additionally, intact heart studies enable the detection of multi-cellular pathophysiological processes such as cardiac arrhythmias. Finally, the use of whole hearts enables the measurement of ventricular transmural gradients of repolarization which play an important role in the pathogenesis of cardiac arrhythmias (Killeen *et al.* 2007, Stokoe *et al.* 2007a,b, Thomas *et al.* 2007a,b). Studies in the whole heart have the advantage of containing all myocardial cell types whilst maintaining intercellular coupling, and could provide more physiologically relevant information regarding the induction and propagation of EADs and arrhythmias. The present study accordingly used a recently established murine whole heart model of arrhythmogensis (Killeen *et al.* 2007), which elicits prominent EADs, triggered activity and VT in the setting of AP prolongation.

We sought to resolve the causal relationship between two macroscopic pathophysiological phenomena, EADs and arrhythmogenic substrate of transmural gradients of repolarization in the genesis of arrhythmias at the whole heart level. The two procedures used nifedipine and KN-93 empirically as pharmacological tools with independent targets to separate out EADs from arrhyrhmogenic substrate, and to assess the contribution of each of these phenomena to the genesis of arrhythmias. In spontaneously beating hearts mid-range concentrations of nifedipine (100 nm) reduced EADs and episodes of VT, but did not alter epicardial or endocardial APD, preserving arrhythmogenic substrate of ΔAPD_90_. Programmed electrical stimulation (PES) applied premature S2 stimuli acting as surrogate EADs and initiated VT in hearts treated with 100 nm nifedipine. A higher concentration of nifedipine (1 μm) similarly eliminated EADs and episodes of VT in spontaneously beating hearts, but also prevented PES-induced VT. These latter effects were accounted for by selective abbreviation of epicardial APD, restoring ΔAPD_90_ to control values and thus removing arrhythmogenic substrate. We corroborated these findings using KN-93, a different pharmacological agent which also affects Ca^2+^ homeostasis through distinct cellular targets compared with nifedipine. KN-93 (2 μM) reduced EADs and episodes of VT in spontaneously beating hypokalaemic hearts but failed to prevent PES-induced VT. Additionally, it was shown that KN-93 did not alter epicardial or endocardial APD and ΔAPD_90_, thus preserving arrhythmogenic substrate.

These results demonstrate the importance of both EADs and arrhythmogenic substrate in the initiation of ventricular arrhythmias at the whole heart level. In the presence of a substrate, an EAD will initiate VT. However, if EADs are empirically suppressed through the use of nifedipine or KN-93 in spontaneously beating hearts, arrhythmias are not initiated. Nevertheless, if the underlying arrhythmic substrate prevails, premature stimuli applied during PES act as surrogate EADs and successfully initiate VT. At the highest concentration used, nifedipine (1 μm) suppressed all EADs and episodes of VT in spontaneously beating hearts, and also selectively abbreviated epicardial APD, restoring ΔAPD_90_ to control levels, thus removing the arrhythmic substrate. Under these conditions PES failed to induce VT. It was thus possible to separate the occurrences or otherwise of EADs, arrhythmic substrate and VT through their differential sensitivities to nifedipine. These findings show for the first time in *any* mammalian cardiac preparation the pharmacological separation of EADs from an arrhythmic substrate, demonstrating the importance of both EADs and arrhythmic substrate in the initiation of arrhythmias at the whole heart level.

## Methods

### Experimental animals

The mice used in this study were kept in an animal house at room temperature and subjected to a consistent 12 h : 12 h light : dark cycle and fed with sterile rodent chow, having access to water at all times. Wild-type (WT) 129 background male and female mice aged 5–7 months were used in all experiments.

### Langendorff-perfused preparation

The experiments used a Langendorff-perfused preparation that has been previously adapted for murine hearts (Balasubramaniam *et al.* 2004). Briefly, mice were killed by cervical dislocation in accordance with schedule 1 of the UK Animals (Scientific Procedures) Act 1986. The heart was then quickly excised and submerged in ice-cold bicarbonate-buffered Krebs–Henseleit solution containing in mm: 119 NaCl, 25 NaHCO_3_, 4 KCl, 1.2 KH_2_PO_4_, 1 MgCl_2_, 1.8 CaCl_2_, 10 glucose and 2 sodium pyruvate. The solution was bubbled with a 95% O_2_–5% CO_2_ mixture (British Oxygen Company, Manchester, UK). The aorta was cannulated under the buffer surface using a 21-gauge custom-made cannula, and was attached to the cannula needle using a micro aneurysm clip (Harvard Apparatus, Edenbridge UK). The preparation was then transferred to the perfusion apparatus, to which the cannula was attached, and perfusion commenced in a retrograde manner via the aorta with the abovementioned bicarbonate-buffered Krebs–Henseleit solution. Before entering the aorta, buffer was passed through 200 μm and 5 μm filters (Milipore, Watford, UK) and warmed to 37 °C by means of a water jacket and circulator (Techne model C-85A, Cambridge, UK). Perfusion was maintained at a constant flow rate of 2 to 2.5 mL min^−1^ using a peristaltic pump (Watson-Marlow Bredel pumps model 505S; Falmouth, Cornwall, UK). Following the start of perfusion, healthy, experimentally viable hearts regained a pink colouration and spontaneous rhythmic contraction with warming. In 10% of experiments, hearts were discarded due to signs of ischaemia after cannulation and perfusion.

### Perfused heart electrophysiological measurements

In the present experiments a paired (1 mm inter-pole spacing) platinum stimulating electrode was placed on the basal surface of the right ventricular epicardium. Before experimental procedures, hearts were paced for 10 min at 8 Hz using 2 ms square-wave stimuli with amplitudes set to three times the excitation threshold (Grass S48 stimulator; Grass-Telefactor, Slough, UK).

Epicardial MAP recordings were obtained using a MAP electrode (Linton Instruments, Harvard Apparatus) placed on the basal surface of the left ventricular epicardium. The epicardial MAP electrode was gradually positioned until a gentle but stable contact pressure was achieved. This resulted in a recording of MAP signals. For endocardial recordings, a small access window was created in the interventricular septum to gain access to left ventricular endocardium (Casimiro *et al.* 2001). A custom-made endocardial MAP electrode constructed from two twisted strands of Teflon-coated (0.25 mm diameter) silver wire (99.99% purity) (Advent Research Materials, Oxford, UK) that had been previously galvanically chlorided to eliminate DC offset, was positioned on to the left ventricular free wall under a stable contact pressure until MAP signals were achieved. MAPs were amplified, band-pass filtered (0.5 Hz to 1 kHz: Gould 2400S; Gould-Nicolet Technologies, Ilford, Essex, UK) and digitized (1401 plus MKII; Cambridge Electronic Design, Cambridge, UK). MAPs were extracted and analysed (Spike II version 4; Cambridge Electronic Design) to derive the precise duration of the digitized signals. The recordings were deemed reproducible and, hence of an acceptable standard for analysis if they had the following properties: a stable baseline, a rapid upstroke phase with consistent amplitude, a smooth contoured repolarization phase and a stable duration [MAP duration at 90% repolarization (APD_90_) was reproducible within 3 ms under baseline conditions].

### Experimental protocol

A standard pacing protocol (basic cycle length, BCL of 125 ms) that corresponded to physiological whole-animal heart rates (Papadatos *et al.* 2002) was initiated for periods of up to 20 min to measure APD at 50%, 70% and 90% repolarization. External pacing stimuli were subsequently withdrawn from all preparations, leading to a significantly reduced, intrinsic heart rate corresponding to a BCL of approximately 400 ms. Reduced heart rates are a known risk factor for the development of repolarization abnormalities such as EADs and triggered beats that may underlie the induction of VT (Roden & Hoffman 1985). Epicardial MAPs were recorded for periods of up to 20 min from isolated, perfused WT mouse hearts under intrinsic pacing conditions. Following this, PES of the heart was carried out using an adaptation of the corresponding clinical techniques (Saumarez & Grace 2000, Balasubramaniam *et al.* 2004). PES procedures began by applying standard pacing stimuli at a BCL of 125 ms for 25 s. Following this, a drive train of eight paced beats (S1) again at a BCL of 125 ms preceded an extrastimulus (S2) every ninth beat. S1S2 intervals initially equalled the pacing interval and then were progressively reduced by 1 ms with each nine beat cycle until ventricular refractoriness was reached, at which point the S2 stimulus elicited no MAP. Recordings were subsequently repeated following a 20 min wash-in of a reduced [K^+^]_o_ perfusate, of 3 mm in the absence and presence of nifedipine (10 nm – 1 μm) or KN-93 (2 μm).

Repolarization time is obtained by the addition of local activation times to APD. Activation time is the time measured from the point of electrical stimulus to the maximal amplitude of the action potential. However, in the present study, we only observed insignificant changes in local activation time whether in the presence of reduced [K^+^]_o_ alone or in combination with any of the pharmacological agents used (data not shown). This is in keeping with a previous study in which perfusion of isolated rabbit hearts with amiodarone led to no significant increase or decrease in local activation times (Kirchhof *et al.* 2003). With this in mind, the present experiments quantified changes in transmural gradients of repolarization by calculating the difference between the epicardial APD_90_ and the endocardial APD_90_. ΔAPD_90_ was calculated from the difference between the mean endocardial and epicardial APD_90_ values, giving positive results where the endocardial value exceeded the epicardial value, and negative results where the epicardial value was greater. An EAD was defined as a positive deflection that interrupted the smooth repolarization phase of the AP. A triggered beat was similarly described as a positive deflection in the smooth repolarization phase of the action potential whose amplitude approximately matched the amplitude of the initial action potential. Arrhythmias were defined as ventricular tachyarrhythmias of more than five cycle duration that were typically self-terminating.

### Experimental solutions

Nifedipine (Sigma, Poole, UK) was initially prepared as a 1 mm stock solution in 96% ethanol. Subsequent dilutions were made in the hypokalaemic buffer solution. All nifedipine solutions were kept wrapped in foil to prevent light degradation. KN-93 (Tocris, Bristol, UK) was initially prepared as a 10 mm stock solution in DMSO, with further dilutions made in the hypokalaemic buffer solution. Final experimental solutions of KN-93 contained no >0.02% DMSO, which had no discernable effects upon MAP morphology in a series of preliminary vehicle control experiments (data not shown).

### Statistical analysis

MAP data were initially imported into Microsoft excel. All data are expressed as mean values ± SEM. Comparisons were made using anova (spss software) with *P*-values <0.05 being considered significant.

## Results

Hypokalaemia is known to predispose to the induction of a lethal ventricular arrhythmia termed TdP (Berthet *et al.* 1999). Several lines of clinical and experimental evidence implicate EADs and triggered activity in the genesis of VT and TdP (Cosio *et al.* 1991, Shimizu *et al.* 1995, Aiba *et al.* 2005, Zhang *et al.* 2005). A recent report has described an arrhythmic model in which isolated murine hearts were perfused with a hypokalaemic physiological buffer solution of 3 mm [K^+^]_o_: the latter procedure results in the preferential prolongation of epicardial over endocardial APD, to give preparations with reduced left ventricular transmural gradients of repolarization, and frequent EADs. This yielded arrhythmic preparations showing triggered activity and episodes of non-sustained VT that thereby fully recapitulated the human clinical phenotype (Killeen *et al.* 2007).

We accordingly used this recently reported murine model of arrhythmogenesis to resolve the relationship between two macroscopic pathophysiological phenomena, EADs and arrhythmogenic substrate of transmural gradients of repolarization in the genesis of arrhythmias at the whole heart level using two independent pharmacological agents that affect Ca^2+^ homeostasis. The experiments recorded left ventricular epicardial and endocardial monophasic action potentials (MAPs) from isolated, perfused murine whole heart preparations under hypokalaemic conditions and following perfusion with nifedipine or KN-93.

### Effects of nifedipine on EADs and spontaneous arrhythmogenesis in intrinsically beating hypokalaemic hearts

Following cannulation and perfusion the electrophysiological parameters of MAP waveform morphology, amplitude and duration reached a steady state within 10 min. MAP recordings in spontaneously beating murine hearts subsequently remained highly reproducible throughout the duration of all experiments. Bradycardia is a recognized risk factor for the development of TdP (Roden & Hoffman 1985) and earlier studies have reported a higher prevalence of EADs and associated VT under bradycardic conditions (Milberg *et al.* 2002, Fabritz *et al.* 2003, Killeen *et al.* 2007).

**Figure 1:**
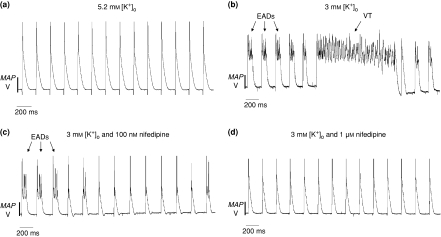
Representative left ventricular intrinsic epicardial monophasic action potential (MAP) recordings from isolated, Langendorff-perfused mouse hearts under control conditions (a), and following perfusion with 3 mm [K^+^]_o_ hypokalaemic solutions in the absence (b) and presence of 100 nm (c) and 1 μm (d) nifedipine. Perfusion of hypokalaemic hearts with 100 nm nifedipine significantly reduced the occurrence of early afterdepolarizations (EADs) and ventricular tachycardia (VT) in six of six hearts. Perfusion with 1 μm nifedipine eliminated EADs and VT in six of six hearts.

To increase the probability of EADs and their associated triggered arrhythmic activity in murine hearts, we recorded left ventricular epicardial and endocardial MAPs from *spontaneously beating* isolated, perfused murine hearts. Spontaneously beating hearts, in the absence of extrinsic pacing, indeed displayed significantly reduced heart rates compared with preparations that were subjected to an extrinsic pacing protocol [spontaneous ventricular CL 334 ± 34 ms (*n* = 6) vs. 125 ms (*n* = 6 paced preparations), *P* < 0.05]. Perfusion with hypokalaemic solutions, nifedipine or KN-93 had no effect upon intrinsic or paced heart rates. Although we were not concerned with the mechanical properties of the murine hearts, we carefully monitored all hearts throughout all experiments to ensure that they were beating continuously. Spontaneously beating hearts perfused with normokalaemic physiological buffer solution in the absence of any pharmacological agents elicited typical murine ventricular MAPs lacking repolarization abnormalities (Fig. 1a). However, following the transition to hypokalaemic conditions, spontaneously beating preparations elicited prominent EADs and triggered beats that preceded episodes of non-sustained VT (*n* = 6; Fig. 1b). EADs were observed in 62.1 ± 7.7% of MAPs recorded from all hypokalaemic hearts in a total recording time in excess of 5 h duration (*n* = 6). Episodes of spontaneous, non-sustained VT were associated with 19.1 ± 5.9% of MAPs recorded from six hypokalaemic preparations over a similar recording period exceeding 5 h duration. We proceeded to investigate the anti-arrhythmic effects of a range of nifedipine concentrations in spontaneously beating hypokalaemic hearts to establish a correlation between EADs and arrhythmogenesis. The specific L-type Ca^2+^ channel LTCC blocking properties of the dihydropyridine, nifedipine are well established (Verheijck *et al.* 1999).

Perfusion of hypokalaemic hearts with 10 nm nifedipine, led to no significant decrease in the incidence of *both* EADs and episodes of VT. In six such separate preparations, 66.9 ± 15.7 and 10.8 ± 6.3% of MAPs were associated with EADs and episodes of VT, respectively (*P* > 0.05). However, perfusion of hypokalaemic hearts with 100 nm nifedipine significantly halved the occurrence of EADs and VT to 28.3 ± 8.7 and 1.2 ± 0.7%, respectively, in six separate preparations (*P* < 0.05, *n* = 6) (Fig. 1c). Finally, perfusion of spontaneously beating isolated hearts with a hypokalaemic solution containing 1 μm nifedipine elicited ventricular MAPs lacking any repolarization abnormalities, such as EADs and triggered beats, in six of six preparations (Fig. 1d). No episodes of spontaneous VT were recorded in *any* of the preparations perfused with a hypokalaemic solution containing 1 μm nifedipine. Nifedipine (100 nm and 1 μm) exerted its anti-arrhythmic effects in hypokalaemic murine hearts immediately following perfusion. A significant suppression of EADs, triggered beats and VT occurred within 30 s.

These data suggest that Ca^2+^ influx through LTCCs is an important factor in the initiation of EADs and of subsequent ventricular arrhythmias in the hypokalaemic murine heart, findings supported by an earlier study in the feline ventricular wedge preparation pharmacologically made to model acquired LQT syndrome, in which the phenylalkylamine LTCC blocker verapamil suppressed EADs and VT (Aiba *et al.* 2005). Nifedipine (100 nm) led to a significant reduction in the occurrence of EADs and VT. Furthermore, this concentration of nifedipine closely matched the IC_50_ for nifedipine at the LTCC previously reported at the single cell level (Shen *et al.* 2000) and our recent findings in which we calculated the IC_50_ for nifedipine in reducing EADs in left ventricular MAPs in a genetically modified mouse model of LQT 3 syndrome to be 79.3 nm (Thomas *et al.* 2007a). At the highest concentration tested, nifedipine (1 μm) eliminated all EADs and episodes of VT in six of six hearts. We proceeded to investigate the anti-arrhythmic effects of nifedipine upon provoked ventricular arrhythmias in hypokalaemic hearts.

### Effects of nifedipine on provoked arrhythmogenesis in hypokalaemic hearts

**Figure 2:**
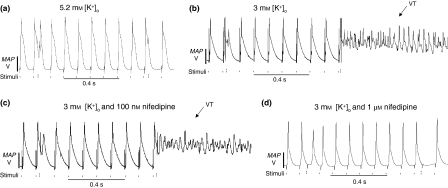
Programmed electrical stimulation (PES) of isolated, Langendorff-perfused mouse hearts under control conditions (a), and following perfusion with 3 mm [K^+^]_o_ hypokalaemic solutions in the absence (b) and presence of 100 nm (c) and 1 μm (d) nifedipine. Ventricular tachycardia (VT) persisted in six of six hypokalaemic hearts perfused with 10 and 100 nm. PES repeatedly failed to induce VT under control conditions (6/6 hearts) and in hypokalaemic hearts perfused with 1 μm nifedipine (6/6 hearts).

Programmed electrical stimulation was next used to determine the arrhythmic susceptibility produced by extrasystolic stimulation of hypokalaemic isolated murine hearts perfused with graded concentrations of nifedipine. In all preparations perfused with control normokalaemic solutions PES failed to induce VT (Fig. 2a). In contrast, closely coupled extra stimuli successfully and reproducibly induced non-sustained VT in six of six hypokalaemic preparations (Fig. 2b). PES-induced VT persisted in hypokalaemic hearts perfused with 10 and 100 nm nifedipine (Fig. 2c) (*n* = 12). However, PES failed to induce VT in any of the six preparations perfused with 1 μm nifedipine (Fig. 2d).

The present results using PES thus demonstrated that only the highest concentration of nifedipine tested (1 μm) protected hypokalaemic preparations against provoked arrhythmogenesis, highlighting a contrast between the PES results and recordings taken from spontaneously beating hearts in which 100 nm nifedipine exerted a significant anti-arrhythmic effect, despite the fact that identical preparations were used in both sets of experiments. We therefore hypothesized that these contrasting anti-arrhythmic effects of 100 nm nifedipine may be due to the fact that 100 nm nifedipine *only* eliminates the triggering factor (EAD) in spontaneously beating hearts, thus preventing VT in this situation but not the arrhythmogenesis provoked by imposed extrasystolic stimulation duing PES. Thus, PES applies early S2 stimuli which may act as surrogate EADs and initiate VT in conditions where an arrhythmic substrate prevails. Such a hypothesis would then require the highest concentration of nifedipine tested (1 μm) to exert *additional* anti-arrhythmic effects beyond suppression of EAD induction, such as alterations in epicardial and endocardial APD which may further reduce arrhythmic propensity in hypokalaemic hearts by reducing arrhythmogenic *substrate* of the transmural gradient of repolarization. Accordingly, we studied the effects of the same concentrations of nifedipine on epicardial and endocardial APD in control and hypokalaemic hearts. Such measurements allow for the quantification of the murine ventricular transmural gradient of repolarization which has been previously correlated with arrhythmogenesis in recent reports (Killeen *et al.* 2007, Stokoe *et al.* 2007a,b, Thomas *et al.* 2007a,b).

### Effect of nifedipine on action potential waveform under hypokalaemic conditions

Epicardial and endocardial APD was measured under steady state extrinsic pacing that corresponded to whole-animal heart rates (Papadatos *et al.* 2002) to eliminate any intrinsic variability in heart rate (Thomas *et al.* 2007a,b). The experiments first measured APD from hearts perfused with normokalaemic and hypokalaemic solutions and in doing so confirmed the recently reported differential effects of hypokalaemia on murine epicardial and endocardial APD and consequently the net transmural gradient of repolarization in the left ventricle (Killeen *et al.* 2007).

**Figure 3:**
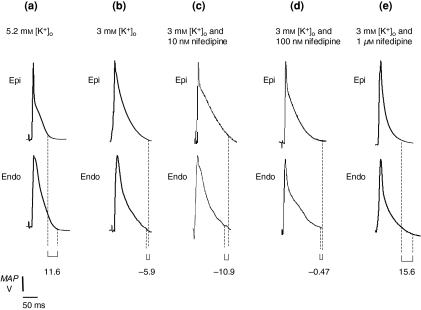
Representative monophasic action potential (MAP) recordings from the left ventricular epicardium and endocardium and transmural gradients of repolarization (endocardial APD_90_ minus epicardial APD_90_) from isolated, Langendorff-perfused mouse hearts during a standard pacing protocol at a basic cycle length of 125 ms under control conditions (a), and following perfusion with 3 mm [K^+^]_o_ hypokalaemic solutions in the absence (b) and presence of 10 nm (c), 100 nm (d) and 1 μm (e) nifedipine.

**Figure 4:**
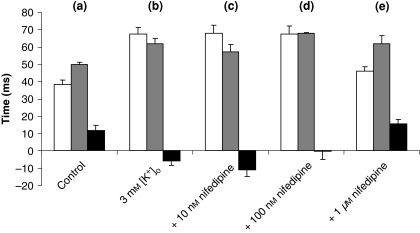
Steady-state epicardial and endocardial APD measured at 90% repolarization (APD_90_), and ΔAPD_90_ values (white, grey and black columns, respectively) under control conditions (a), and following perfusion with 3 mm [K^+^]_o_ hypokalaemic solutions in the absence (b) and presence of 10 nm (c), 100 nm (d) and 1 μm (e) nifedipine.

Contrasting APD measurements between the epicardium and the endocardium were found in hypokalaemic preparations. Hearts perfused with normokalaemic physiological buffer solution in the absence of any pharmacological agents elicited typical murine ventricular epicardial and endocardial MAPs: a triangular morphology and a smooth repolarization phase lacking repolarization abnormalities such as EADs or triggered beats (Fig. 3a). Endocardial APD_90_ was significantly greater than epicardial APD_90_ (50.0 ± 1.4 vs. 38.4 ± 2.4 ms, respectively, *n* = 6, *P* < 0.05) giving a corresponding ΔAPD_90_ of 11.6 ± 3.0 ms (Figures 3a and 4a). Perfusion of isolated hearts with 3 mm [K^+^]_o_ preferentially prolonged epicardial compared with endocardial APD at 90% repolarization time (APD_90_) (Fig. 3b). Epicardial APD_90_ was increased from 38.4 ± 2.4 to 66.1 ± 3.4 ms and endocardial APD_90_ was increased to 50.0 ± 1.4 to 62.6 ± 3.6 ms (*P* < 0.05, *n* = 6) (Figs 3b and 4b). We documented preferential epicardial vs. endocardial action potential prolongation, insofar as epicardial APD_90_ exceeded endocardial APD_90_ under hypokalaemic conditions. These effects led to a dramatic reduction in the transmural gradient of repolarization, ΔAPD_90_. Under these hypokalaemic conditions, ΔAPD_90_ was significantly altered from 11.6 ± 3.0 ms to −5.9 ± 2.5 ms (Figs 3a and 3b, 4a and b) (*P* < 0.05, *n* = 6), confirming our earlier findings (Killeen *et al.* 2007).

We then proceeded to investigate the effects of nifedipine at a range of concentrations (10 nm, 100 nm and 1 μm) upon hypokalaemia-induced action potential prolongation at epicardial and endocardial left ventricular sites. Perfusion of hypokalaemic hearts with 10 nm nifedipine did not significantly alter epicardial or endocardial APD_90_ (68.0 ± 4.6 and 57.1 ± 4.5 ms, respectively; *n* = 6, *P* > 0.05) (Figs 3c and 4c); similar findings resulted from perfusion with 100 nm nifedipine (67.6 ± 0.3 and 68.0 ± 4.6 ms, respectively; *n* = 6, *P* > 0.05) (Figs 3d and 4d). Consequently, the transmural gradient of repolarization was unaltered in the presence of 10 and 100 nm nifedipine, with ΔAPD_90_ values, −10.9 ± 3.7 and −0.47 ± 4.4 ms, respectively, which were not significantly different to baseline hypokalaemic values (*n* = 12) (Fig. 4c and d). However, at the highest concentration tested, nifedipine (1 μm) selectively abbreviated epicardial APD_90_ under hypokalaemic conditions to 46.2 ± 2.5 ms (*P* < 0.001), whilst preserving endocardial APD_90_ (61.8 ± 5 ms; *P* > 0.05), resulting in the normalization of the transmural gradient of repolarization, as reflected in a ΔAPD_90_ 15.5 ± 3.2 ms, restoring the expected pattern of murine ventricular repolarization in which endocardial APD is greater than epicardial APD (Figs 3e and 4e). Under control normokalaemic conditions and under hypokalaemic conditions in the presence of 1 μm nifedipine, ΔAPD_90_ and values were statistically insignificant from one another (*P* > 0.05).

To determine if these effects of 1 μm nifedipine upon epicardial APD, and hence the transmural gradient of repolarization were dependant on the setting of hypokalaemia, we perfused hearts with a normokalaemic physiological solution containing 1 μm nifedipine. In six separate preparations under normokalaemic conditions, 1 μm nifedipine did not significantly alter epicardial or endocardial APD from control values (42.2 ± 2.6 and 50.8 ± 3.0 ms, respectively, *P* > 0.05, *n* = 6). Accordingly in hearts perfused with 1 μm nifedipine under normokalaemic conditions ΔAPD_90_ was not significantly altered from control normokalaemic values (8.6 ± 4.0 vs. 11.6 ± 3.0 ms, respectively, *P* > 0.05, *n* = 6). These data suggest that the effects of nifedipine upon epicardial APD are exclusive to a hypokalaemic state.

Perfusion of hypokalaemic hearts with 1 μm nifedipine restored ΔAPD_90_ to a positive value indistinguishable from control values, responses never recorded following perfusion with 10 and 100 nm nifedipine in which a negative ΔAPD_90_ value prevailed. Selective abbreviation of epicardial over endocardial APD in hypokalaemic hearts by 1 μm nifedipine accounted for this phenomenon and supported earlier findings using verapamil in the feline ventricular wedge preparation made to model acquired LQT syndrome (Aiba *et al.* 2005). We have previously correlated negative values of ΔAPD_90_ with ventricular arrhythmias in a range of murine models of arrhythmogenesis including hypokalaemia-induced VT (Killeen *et al.* 2007) and genetically modified models of LQT3 (Stokoe *et al.* 2007a,b, Thomas *et al.* 2007a) and LQT5 (Thomas *et al.* 2007b). In keeping with these studies, perfusion of hypokalaemic hearts with 1 μm nifedipine restored ΔAPD_90_ to positive values and was associated with a marked reduction in arrhythmogenecity under both spontaneously beating and PES protocols. These data suggest that arrhythmic substrate of ΔAPD_90_ is only affected by 1 μm nifedipine.

### Effects of alteration of calcium homeostasis through inhibition of calmodulin kinase type II

Calmodulin kinase type II (CaMKII) has been shown to be a proarrhythmic signalling molecule in an experimental model of drug-induced LQT syndrome (Wu *et al.* 1999). We accordingly assessed for the first time the effects of acute pharmacological inhibition of CaMKII with KN-93, a direct inhibitor of CaMKII (Fleming *et al.* 1998) in establishing a causal relationship between EADs and arrhythmic substrate in the initiation of arrhythmias at the whole heart level for the first time in any cardiac preparation.

**Figure 5:**
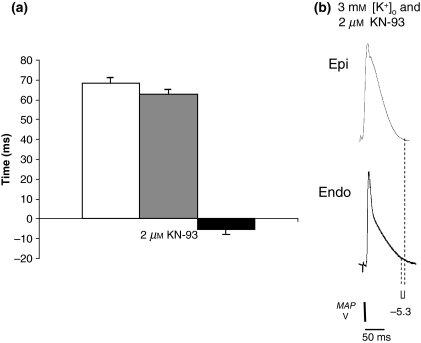
Steady-state epicardial and endocardial APD measured at 90% repolarization (APD_90_), and ΔAPD_90_ values (white, grey and black columns, respectively) (a) and representative monophasic action potential (MAP) recordings from the left ventricular epicardium and endocardium and transmural gradient of repolarization (b) from isolated, Langendorff-perfused wild-type (WT) mouse hearts perfused with 3 mm [K^+^]_o_ hypokalaemic solutions in the presence of 2 μm KN-93.

The concentration of KN-93 used in the present study was based upon a careful consideration of the literature. Previously, at the single cell level, CaMKII inhibitors have been used at concentrations ranging from 1 μm (Wu *et al.* 2002) to 20 μm (Wu *et al.* 1999). An additional six hypokalaemic hearts were perfused with KN-93 (2 μm). Firstly, under hypokalaemic conditions, KN-93 did not significantly alter epicardial or endocardial APD_90_ (Fig. 5a and b) (68.3 ± 2.7 and 62.9 ± 2.0 ms, respectively; *P* > 0.05, *n* = 6). Accordingly, the transmural gradient of repolarization, reflected in ΔAPD_90_, was not significantly different to values under hypokalaemic conditions (Fig. 5a and b) (−5.3 ± 2.8 vs. −5.9 ± 2.5 ms, respectively, *P* > 0.05, *n* = 6).

**Figure 6:**
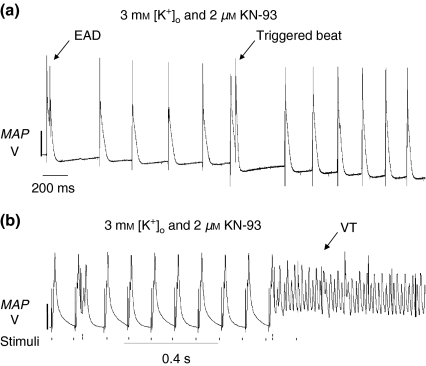
Representative left ventricular intrinsic epicardial monophasic action potential (MAP) recordings (a), and programmed electrical stimulation (PES) (b) of mouse hearts perfused with 3 mm [K^+^]_o_ hypokalaemic solutions in the presence of 2 μm KN-93. KN-93 reduced the occurrence of early afterdepolarizations (EADs), triggered beats and ventricular tachycardia (VT) in spontaneously beating hearts but failed to protect against provoked arrhythmogenesis during PES in six of six hearts.

Secondly, spontaneously beating hypokalaemic hearts perfused with KN-93 elicited a significantly reduced frequency of both EADs and episodes of VT. In a total of six separate preparations throughout a total recording time in excess of 5 h duration, the percentage of MAPs associated with EADs was significantly reduced from 62.1 ± 7.7% under hypokalaemic conditions to 29.6 ± 8.9% following perfusion with KN-93 (*P* < 0.05, *n* = 6) (Fig. 6a). Similarly, the percentage of MAPs associated with episodes of non-sustained VT significantly fell from 19.1 ± 5.9% under hypokalaemic conditions, to 1.7 ± 1.1% in the presence of KN-93 over a similar length of recording time in six separate preparations (*P* < 0.05, *n* = 6).

Finally, the anti-arrhythmic efficacy of KN-93 in the setting of hypokalaemia was quantified by applying PES procedures. PES induced VT in six of six hypokalaemic preparations perfused with KN-93 (Fig. 6b). Whereas 2 μm KN-93 significantly reduced arrhythmogenecity in spontaneously beating hypokalaemic hearts, it failed to protect against provoked arrhythmogenesis under PES protocols in six of six hypokalaemic hearts. KN-93 did not have any effect upon epicardial or endocardial APD, and the resulting ΔAPD_90_ value was not significantly different compared with baseline hypokalaemic hearts. Thus PES applies early S2 stimuli, acting as surrogate EADs, upon an arrhythmogenic substrate of ΔAPD_90_ which induced VT. Collectively these findings demonstrate for the first time the pharmacological separation of EADs and altered transmural gradients of repolarization in a whole heart model of arrhythmogenesis through the application of two independent agents each with different molecular targets.

## Discussion

Hypokalaemia, a common medical condition, is known to have severe consequences in particular clinical situations (Steiness & Olesen 1976) and is a known risk factor for the development of TdP and related, potentially lethal arrhythmias (Berthet *et al.* 1999). EADs and changes in the ventricular transmural gradient of repolarization are known key contributors to the pathogenesis of ventricular arrhythmias (Milberg *et al.* 2005, Thomas *et al.* 2007a,b, Vandenberg *et al*., 2001). We took advantage of a recently developed whole heart model of arrhythmogenesis (Killeen *et al.* 2007) to study for the first time the precise pathophysiological causal relationship between EADs and transmural gradients of repolarization in the development of ventricular arrhythmias at the whole heart level using two independent modifiers of Ca^2+^ homeostasis.

The present study used the contact MAP electrode technique (Franz *et al*., 1999). It was an absolute requirement of our study for the myocardium to be intact, and working under physiological conditions. The use of *any other* technique to measure APs would have required the dissection and preparation of the myocardium into isolated tissue preparations or isolation of the myocardium into single cells. Such procedures remove re-entry mechanisms, intercellular coupling and dispersions of repolarization present in the whole heart, which are all considered to play a crucial role in arrhythmogenesis. Transmembrane APs (TAPs) require impalement of cardiac tissue with a sharp glass microelectrode, which negates their use in the recording of *in situ* waveforms from the beating heart. MAPs are extracellularly recorded waveforms that have been previously shown to accurately reproduce waveform morphology, amplitude and repolarization times of transmembrane action potentials (TAPs) from the mouse heart with a high level of accuracy (Knollmann *et al.* 2001). The durations and transmural dispersion repolarization times we report in the present study under control conditions and following perfusion with hypokalaemic solutions in the absence and presence of nifedipine and KN-93 are statistically significant increases or decreases as opposed to measurements of absolute values, and correspond to previous measurements in earlier studies (Fabritz *et al.* 2003, Killeen *et al.* 2007). These data thus represent highly accurate and reproducible changes that have been recorded from the ventricular surfaces of the intact, beating mouse heart over a considerable length of time demonstrating a high level of stability of our MAP recordings.

Firstly, the present study has shown that nifedipine eliminated EADs and VT in spontaneously beating hypokalaemic hearts in a concentration-dependant fashion. Spontaneously beating hearts proved a particularly appropriate system for such study, as their relatively reduced heart rates provoked preparations with an increased propensity for the development of repolarization abnormalities such as EADs and triggered beats, which preceded episodes of VT in the setting of hypokalaemia (Killeen *et al.* 2007). Furthermore, these findings at the whole heart level correlate with earlier cellular studies which implicated *I*_Ca,L_ as the necessary depolarizing charge carrier for the induction of EADs at slow stimulation rates (Damiano & Rosen 1984, Zeng & Rudy 1995).

The proposed mechanism for EADs in the setting of action potential prolongation is the elevation of intracellular Ca^2+^ concentration through the increased opening of LTCCs that occurs secondary to action potential prolongation, demonstrated in isolated cardiac myocytes. Impaired cardiac repolarization and critical lengthening of APD leads to a lengthening of the cardiac action potential plateau within a critical voltage ‘window’ range enabling reactivation of LTCCs, and a subsequent secondary release of calcium from the sarcoplasmic reticulum (SR) leading to an aftercontraction (January & Riddle 1989, Ming *et al.* 1994, Viswanathan *et al.* 1999). Accordingly, reductions in [K^+^]_o_ have been associated with action potential prolongation and an appearance of EADs and arrhythmogenesis (Killeen *et al.* 2007).

Whereas 10 nm nifedipine had no effects upon the frequency of unprovoked EADs and VT in spontaneously beating hypokalaemic hearts, 100 nm nifedipine significantly halved the occurrence of both EADs and subsequent VT in six of six preparations. Finally, perfusion of hypokalaemic hearts with 1 μm nifedipine eliminated EADs in six of six hearts and abolished VT in all preparations. This finding is in agreement with our recent study in which nifedipine reduced EADs in spontaneously beating mouse hearts genetically modified to model human LQT3 syndrome with an EC_50_ of 79.3 nm (Thomas *et al.* 2007a).

Secondly, in contrast to the above assessments of *spontaneous* arrhythmogenecity, we assessed the tendency to arrhythmogenesis specifically *provoked* by imposed extrasystolic stimulation, and the effect upon this of nifedipine in the hypokalaemic heart at a range of concentrations using an established method of PES. Under control, normokalaemic conditions, VT was never induced in preparations during PES protocols. However, PES successfully induced VT in all hypokalaemic hearts, in addition to those perfused with 10 nm nifedipine. Similarly, 100 nm nifedipine failed to prevent *provoked* ventricular arrhythmogenesis in all six preparations, in contrast to its anti-arrhythmic effects recorded from *spontaneously* beating hypokalaemic hearts. However, PES failed to induce VT in any hypokalaemic preparation perfused with 1 μm nifedipine.

Thirdly, the above contrast suggests that 1 μm nifedipine may be exerting additional effects, beyond suppression of EADs, which could also account for its anti-arrhythmic efficacy in the hypokalaemic heart. We accordingly sought to investigate the basis of these differential efficacies shown by nifedipine by measuring changes in the transmural gradient of repolarization in response to perfusion with increasing concentrations of nifedipine. These explorations were prompted by earlier reports that associated changes in the transmural gradient of repolarization with arrhythmogenecity in a range of cardiac models (Milberg *et al.* 2005, Killeen *et al.* 2007, Stokoe *et al.* 2007a,b, Thomas *et al.* 2007a,b). The present experiments have shown that administration of 1 μm nifedipine to hypokalaemic hearts led to selective attenuation of epicardial as opposed to endocardial APD_90_ in hypokalaemic hearts. This restored the ΔAPD_90_ in hypokalaemic hearts to values seen in non-arrhythmogenic, untreated normokalaemic hearts. This finding is in agreement with our recent study using arrhythmogenic mice modelling human LQT5 syndrome through targeted disruption of KCNE1, in which 1 μm nifedipine selectively reduced epicardial APD, whilst having no similar affect upon endocardial APD (Thomas *et al.* 2007b). In the present study, such findings were not recorded following perfusion with lower concentrations of nifedipine. Additionally, Aiba *et al.* (2005) showed that verapamil preferentially abbreviated epicardial compared with endocardial APD, leading to a normalization in the transmural gradient of repolarization alongside the suppression of EADs, triggered activity and TdP in the perfused feline left ventricle made to model subclinical dysfunction of *I*_Ks_ alongside drug-induced LQTS. These effects of nifedipine upon epicardial APD appeared to be exclusive to the pathophysiological state of hypokalaemia. Thus under normokalaemic conditions the highest concentration of nifedipine had no effects upon epicardial and endocardial APD and hence ΔAPD_90_ in wild-type hearts, in keeping with our recent findings (Thomas *et al.* 2007b). In the present study, we report for the first time that epicardial APD is reduced by the LTCC blocker nifedipine under hypokalaemic conditions at micromolar concentrations.

At the whole heart level, we have previously shown that nifedipine (1 μm) eliminated episodes of VT in genetically modified mouse models of LQT3 and LQT5 generated through targeted disruption of SCN5a and KCNE1, respectively (Thomas *et al.* 2007a,b) through blockade of the LTCC. However, nifedipine may also exert its anti-arrhythmic efficacy through reduced SR calcium release secondary to longer inhibition of calcium influx through LTCCs. Indeed, we have previously shown that pre-treatment of isolated murine ventricular myocytes with nifedipine reduces electrically evoked calcium transients, indicative of a reduction in SR calcium release (Balasubramaniam *et al.* 2004). Nevertheless due to the rapid onset of action of nifedipine in the hypokalaemic murine heart, we believe that *acute* inhibition of the L-type calcium channel by nifedipine predominantly accounts for its anti-arrhythmic efficacy. In the present study, we were concerned primarily with the elucidation of the electrical abnormalities in the intact heart that underlie arrhythmogenesis – EADs and altered transmural gradients of repolarization. We were not concerned with the contractile or mechanical properties of the heart. However, a reduction in inotropy and hence cardiac output through LTCC blockade may also account for the anti-arrhythmic effects of nifedipine. However, a previous study has determined that inhibition of EADs and VT by nifedipine in the rabbit heart significantly preceded any discernable decreases in left ventricular developed pressure (LVDP) (Anderson *et al.* 1998). Taken together these findings support the notion that the anti-arrhythmic effects induced through pharmacological inhibition of the LTCC by nifedipine occur *independently* of significant effects upon the mechanical functions of the isolated murine heart.

We used maximum nifedipine concentrations of 1 μm in the present experiments; concentrations between 1 and 2 μm suffice to substantially reduce Ca^2+^ transients and *I*_Ca,L_ in isolated murine and rabbit myocytes (Balasubramaniam *et al.* 2004). Furthermore, nifedipine concentrations as high as 5 μm have no effects upon *I*_Ca,T_, *I*_Na_, *I*_K_ and *I*_f_ (Verheijck *et al.* 1999, Gao *et al.* 2005). Additionally in isolated rodent myocytes, the inwardly rectifying current (*I*_K1_), the rapidly activating delayed-rectifier current (*I*_Kr_) and slowly activating delayed-rectifier current (*I*_Ks_) are unaffected by nifedipine concentrations ≤10 μm, although individual currents were affected by nifedipine concentrations of 260, 275 and 360 μm respectively (Zhabyeyev *et al.* 2004). Whatever the underlying mechanism governing the anti-arrhythmic efficacy of nifedipine, these results using a range of concentrations of nifedipine empirically demonstrate for the first time the pharmacological separation of EADs from arrhythmic substrate using a modifier of Ca^2+^ homeostasis in a whole heart model of arrhythmogenesis. We thus implicate both EADs and arrhythmic substrate in the induction of arrhythmias at the whole heart level.

Finally, we corroborated these findings with nifedipine using an independent pharmacological agent which also affects Ca^2+^ homeostasis through inhibition of CaMKII, KN-93. We report that KN-93 indeed leads to a significant reduction of EADs, triggered beats and episodes of VT in the spontaneously beating hypokalaemic heart. In the present study, KN-93 significantly reduced the frequency of EADs and episodes of VT in spontaneously beating hypokalaemic hearts, yet failed to protect against provoked arrhythmogenesis in the PES studies. Furthermore, KN-93 failed to significantly alter epicardial or endocardial APD_90_ under hypokalaemic conditions, preserving arrhythmogenic substrate of ΔAPD_90_. Thus, PES induced early stimuli, acting as surrogate EADs, imposed upon an arrhythmogenic substrate of ΔAPD_90_ to induce VT. To the best of our knowledge this is the first report documenting these differential anti-arrhythmic effects of CaMKII inhibition in any cardiac preparation.

Calmodulin kinase type II has emerged as an important arrhythmogenic signalling molecule in the setting of LQT syndrome (Wu *et al.* 1999), cardiac hypertrophy (Wu *et al.* 2002) and cardiomyopathy (Khoo *et al.* 2005). Following initial activation by increased [Ca^2+^]_*i*_, CaMKII activity becomes partly Ca^2+^-independent, through a mechanism involving intersubunit autophosphorylation (Braun & Schulman 1995). In arrhythmogenic rabbit hearts Anderson *et al.* (1998) measured a significant increase in Ca^2+^-independent CaMKII activity compared with control hearts. Pre-treatment with KN-93 abolished arrhythmogenecity and corresponding increases in Ca^2+^-independent CaMKII activity (Anderson *et al.* 1998). These data suggest that CaMKII activity, although dependant upon [Ca^2+^]_*i*_ for initial activation, transitions into a Ca^2+^-independent state and plays an important role in EAD induction and subsequent arrhythmogenesis.

Studies have ascribed the anti-arrhythmic effects of acute pharmacological inhibition of CaMKII to decreased activity of LTCCs. A study by Wu *et al.* (2004) demonstrated that calmodulin kinase is functionally targeted to and is a critical regulator of the LTCC. Additionally in genetically modified mice modelling cardiac hypertrophy through increased CaMKII activity, Wu *et al.* (2002) showed that transgenic ventricular myocytes had an increased LTCC open probability, compared with WT myocytes, corresponding to a high frequency of EADs and arrhythmias in the transgenic mice. Pharmacological inhibition of CaMKII reduced LTCC open probability in transgenic myocytes to levels found in WT myocytes (Wu *et al.* 2002). Thus, pharmacological inhibition of CaMKII is an effective measure to target the LTCC and to exert potent anti-arrhythmogenic effects.

Additionally, CaMKII has also been shown to affect other ion channels and intracellular targets. The sodium–calcium exchanger (NCX) is regulated by CaMKII activity (Wu *et al.* 1999) which can cause arrhythmogenic inward currents which may give rise to EADs. However, previous studies have shown that selective NCX inhibition does not preclude the induction of EADs and arrhythmias in a range of arrhythmogenic cardiac models (Shinada *et al.* 2005, Zhang *et al.* 2005). It was recently shown that CaMKII may reduce Na^+^ channel availability at high heart rates and increase late Na^+^ current in transgenic mice over expressing CaMKII (Wagner *et al.* 2006). Reduced Na^+^ channel availability would be expected to reduce APD, whereas increased late Na^+^ current would be expected to increase APD. We observed neither an increase nor a decrease in APD in hypokalaemic hearts treated with the CaMKII inhibitor KN-93, precluding an effect of KN-93 on Na^+^ channels in the present study.

A recent study has also suggested that inhibition of CaMKII activity may increase repolarizing K^+^ currents, in particular *I*_to_ (Li *et al*. 2006). We consider these actions unlikely in the present study for the following reasons. Chronic, genetic inhibition of CaMKII was necessary for the observed changes in *I*_to_, acute pharmacological inhibition of CaMKII had no effect upon repolarizing K^+^ currents (Li *et al*. 2006). Furthermore, acute inhibition of CaMKII by KN-93 did not significantly affect epicardial or endocardial APD in the present study. KN-93 has also recently been shown to block K^+^ channels and reduce repolarizing K^+^ currents (Rezazadeh *et al.* 2006). Inhibition of K^+^ channels by KN-93 would be expected to prolong the cardiac AP, which may potentially negate any beneficial anti-arrhythmic effects. Indeed, Kirchhof *et al.* (2004) showed that KN-93 prolonged epicardial APD in mice under control, normokalaemic conditions. However, in the present study, we consider the blocking effects KN-93 upon K^+^ channels unlikely for several reasons. Firstly, KN-93 failed to significantly affect epicardial or endocardial APD in the hypokalaemic heart. Secondly, if KN-93 did block K^+^ channels and prolong APD, one would expect an increased level of EADs and episodes of VT, which we did *not* observe. These findings exclude an effect of KN-93 upon repolarizing K^+^ channels in the present study.

Sarcoplasmic and endoplasmic reticulum ATP-ase (SERCA) activity can also be affected by CaMKII via phosphorylation of phospholamban (Tada *et al.* 1982). Thus, KN-93 inhibition of CaMKII inhibits the phosphorylation of phospholamban and was shown to significantly decrease SR Ca^2+^ content (Kim *et al*. 2000). Additionally, previous studies have documented either increased (Guo *et al.* 2006) or decreased (Wu *et al.* 2002) SR Ca^2+^ release associated with CaMKII activity. Guo *et al.* (2006) demonstrated that endogenous CaMKII activity can phosphorylate the ryanodine receptor (RyR2), leading to an increase in channel opening and increased SR Ca^2+^ release events. Inhibition of CaMKII by KN-93 may thus reduce SR Ca^2+^ content and reduce RyR2 channel opening, effects which may also contribute to its anti-arrhythmic efficacy. Agents which affect Ca^2+^ homeostasis may alter myocardial inotropy and cardiac output, which may also be an anti-arrhythmogenic mechanism of action. However, a study by Valverde *et al.* (2004) concluded that inhibition of CaMKII by KN-93 did not play a significant role in mechanisms governing myocardial contraction and relaxation in the isolated, perfused rat heart.

Nevertheless, we used KN-93 as a tool which affects Ca^2+^ homeostasis to empirically separate out EADs from arrhythmogenic substrate in the hypokalaemic murine heart and to corroborate the findings we observed using nifedipine. Whatever the underlying anti-arrhythmogenic mechanism of KN-93, we have demonstrated that acute inhibition of CaMKII reduces arrhythmogenecity in spontaneously beating hearts by removing the trigger for the arrhythmia, the EAD. The failure of KN-93 to restore ΔAPD_90_, maintains the arrhythmic substrate and leads to the induction of arrhythmias in provoked studies using PES.

In conclusion, we report that the empirical use of two independent pharmacological agents that modify Ca^2+^ homeostasis, nifedipine and KN-93, permit the separation of two of the previously accepted predominantly causative factors for arrhythmogenesis: EADs and arrhythmic substrate. These findings were demonstrated in a recently reported whole heart model of arrhythmogenesis that presents with action potential prolongation, EADs, triggered beats and VT, and which fully recapitulates the human clinical phenotype (Killeen *et al.* 2007). To the best of our knowledge these findings in the present study have not been reported in any other mammalian cardiac preparation. KN-93 and mid-range concentrations of nifedipine eliminated EADs but failed to alter ΔAPD_90_, actions which abolished spontaneously occurring arrhythmias but preserved provoked arrhythmias using PES. Collectively, these findings clarify the causal relationship between EADs and arrhythmic substrate in the induction of arrhythmias at the whole heart level.

## Conflict of interest

No conflict of interest.
